# Genetic and environmental control of host-gut microbiota interactions

**DOI:** 10.1101/gr.194118.115

**Published:** 2015-10

**Authors:** Elin Org, Brian W. Parks, Jong Wha J. Joo, Benjamin Emert, William Schwartzman, Eun Yong Kang, Margarete Mehrabian, Calvin Pan, Rob Knight, Robert Gunsalus, Thomas A. Drake, Eleazar Eskin, Aldons J. Lusis

**Affiliations:** 1 Department of Medicine/Division of Cardiology, David Geffen School of Medicine, University of California, Los Angeles, California 90095, USA;; 2 Bioinformatics IDP, University of California, Los Angeles, California 90095, USA;; 3 Department of Computer Science, University of California, Los Angeles, California 90095, USA;; 4 Department of Human Genetics, David Geffen School of Medicine, University of California, Los Angeles, California 90095, USA;; 5 Departments of Pediatrics and Computer Science and Engineering, University of California, San Diego, California 92093, USA;; 6 Department of Microbiology, Immunology and Molecular Genetics, University of California, Los Angeles, California 90095, USA;; 7 Department of Pathology and Laboratory Medicine, University of California, Los Angeles, California 90095, USA;; 8 Department of Microbiology, Immunology and Molecular Genetics, University of California, Los Angeles, California 90095, USA

## Abstract

Genetics provides a potentially powerful approach to dissect host-gut microbiota interactions. Toward this end, we profiled gut microbiota using 16s rRNA gene sequencing in a panel of 110 diverse inbred strains of mice. This panel has previously been studied for a wide range of metabolic traits and can be used for high-resolution association mapping. Using a SNP-based approach with a linear mixed model, we estimated the heritability of microbiota composition. We conclude that, in a controlled environment, the genetic background accounts for a substantial fraction of abundance of most common microbiota. The mice were previously studied for response to a high-fat, high-sucrose diet, and we hypothesized that the dietary response was determined in part by gut microbiota composition. We tested this using a cross-fostering strategy in which a strain showing a modest response, SWR, was seeded with microbiota from a strain showing a strong response, A×B19. Consistent with a role of microbiota in dietary response, the cross-fostered SWR pups exhibited a significantly increased response in weight gain. To examine specific microbiota contributing to the response, we identified various genera whose abundance correlated with dietary response. Among these, we chose *Akkermansia muciniphila*, a common anaerobe previously associated with metabolic effects. When administered to strain A×B19 by gavage, the dietary response was significantly blunted for obesity, plasma lipids, and insulin resistance. In an effort to further understand host-microbiota interactions, we mapped loci controlling microbiota composition and prioritized candidate genes. Our publicly available data provide a resource for future studies.

Studies carried out over the last decade have revealed that gut microbiota contribute to a variety of common disorders, including obesity and diabetes (Musso et al. 2011), colitis (Devkota et al. 2012), atherosclerosis (Wang et al. 2011), rheumatoid arthritis (Vaahtovuo et al. 2008), and cancer (Yoshimoto et al. 2013). The evidence for metabolic interactions is particularly strong, as a large body of data now supports the conclusion that gut microbiota influence the energy harvest from dietary components, particularly complex carbohydrates, and that metabolites such as the short-chain fatty acids produced by gut bacteria can perturb metabolic traits, including adiposity and insulin resistance (Turnbaugh et al. 2006, 2009; Backhed et al. 2007; Wen et al. 2008; Ridaura et al. 2013). Gut microbiota communities are assembled each generation, influenced by maternal seeding, environmental factors, host genetics, and age, resulting in substantial variations in composition among individuals in human populations (Eckburg et al. 2005; Costello et al. 2009; Human Microbiome Project Consortium 2012; Goodrich et al. 2014). Most experimental studies of host-gut microbiota interactions have employed large perturbations, such as comparisons of germ-free versus conventional mice, and the significance of common variations in gut microbiota composition for disease susceptibility is still poorly understood. Furthermore, while studies with germ-free mice have clearly implicated microbiota in clinically relevant traits, it has proven difficult to identify the responsible taxa of bacteria.

We now report a population-based analysis of host-gut microbiota interactions in the mouse. One of the issues we explore is the role of host genetics. Although some evidence is consistent with significant heritability of gut microbiota composition, the extent to which the host controls microbiota composition under controlled environmental conditions is unclear. We also examined the role of common variations in gut microbiota in metabolic traits such as obesity and insulin resistance and mapped loci contributing to the abundance of certain microbiota. We performed our study using a resource termed the Hybrid Mouse Diversity Panel (HMDP), consisting of about 100 inbred strains of mice that have been either sequenced or subjected to high-density genotyping (Bennett et al. 2010). The resource has several advantages for genetic analysis as compared to traditional genetic crosses. First, it allows high-resolution mapping by association rather than linkage analysis, and it has now been used for the identification of a number of novel genes underlying complex traits (Farber et al. 2011; Lavinsky et al. 2015; Parks et al. 2015; Rau et al. 2015). Second, since the strains are permanent, the data from separate studies can be integrated, allowing the development of large, publicly available databases of physiological and molecular traits relevant to a variety of clinical disorders (systems.genetics.ucla.edu and phenome.jax.org). Third, the panel is ideal for examining gene-by-environment interactions, since it is possible to examine individuals of a particular genotype under a variety of conditions (Orozco et al. 2012; Parks et al. 2013).

## Results

### Variation of gut microbiota in a large panel of mouse strains

We determined the composition and variability of gut microbiota in a total of 599 mice from 113 HMDP strains, of which 327 were male and 273 were female (average three mice per strain) (Supplemental Table 1). All mice in the study were bred for two or more generations in the same facility at UCLA, and each strain was maintained in separate cages (two to four mice of each strain). The mice were maintained on a chow diet (6% kcal from fat) until 8 wk of age, and then placed on a high-fat, high-sucrose (HF/HS) diet for anadditional 8 wk. We performed multiplex 16S rRNA sequencing of the V4 amplicon using the Illumina MiSeq platform. On average, 23,048 reads were obtained per sample (ranging from 6331 to 82,238). Reads were binned into individual samples based on barcode sequence, and complementary taxon-based analysis methods were used to compare 16S rRNA sequences across the cecum microbial communities. The relative abundances of phylum, class, order, family, and genus were determined for the 599 mice. We focused on abundant microbes, defined as operational taxonomic units (OTUs) with at least 0.01% relative abundance across all samples (total 439 OTUs).

We previously showed that changing a chow to a HF/HS diet drastically changed microbiota composition across HMDP strains and that these shifts were strongly dependent on the genetic background of the mice (Parks et al. 2013). After HF/HS diet feeding, we identified 49 genera, where the 17 most abundant genera were present in at least 75% of the samples (*n* = 599). These seventeen most abundant genera accounted for 68% of reads and included members of the six phyla (Supplemental Table 2). Consistent with previous findings in both mice and humans, the most abundant phyla in the gut were Firmicutes (49.8% ± 10.9) and Bacteroidetes (41.8% ± 9.6). Compared to the chow diet, the HF/HS diet resulted in increased Firmicutes and decreased Bacteroidetes, consistent with previous studies (Wu et al. 2011; Carmody et al. 2015).

**Figure 1.**
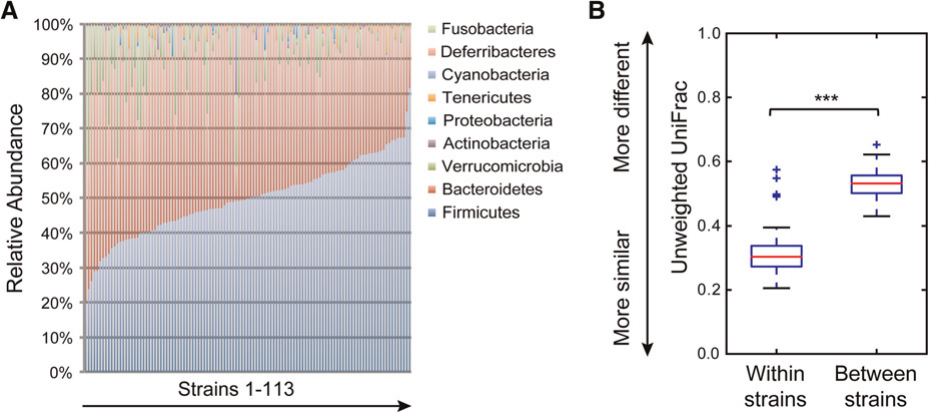
Phylum-level variability of gut microbiota composition across 113 inbred strains of mice. (*A*) Columns represent the relative abundance of microbial phyla in 113 strains (327 male and 297 female). (*B*) Box plot of β diversity distances between microbial communities obtained when comparing mice within and between strains. The specific distance metric used is indicated on the axes. (***) *P* < 0.001 for Student's *t*-test with 1000 Monte Carlo simulations. See also Supplemental Table 2.

Microbiota composition varied greatly across the 110 strains of mice (Fig. 1A; Supplemental Table 2). For instance, the relative abundance of the Firmicutes across all the strains ranged between 20% and 82%. Even larger variations were observed at finer taxonomic levels; for example, a common mucus layer inhabitant in gut, *Akkermansia muciniphila* (*A. muciniphila*), varied in abundance from 0.005% to 40% across the strains of mice. In contrast to human data, we were not able to detect any members of hydrogen-consuming methanogens, although not all methanogens, which are Archaea, would be expected to amplify with bacterial 16S RNA gene primers.

### Heritability estimation of gut microbiota composition

In examining individual mice housed in separate cages (to avoid cage effects since mice are coprophagic), we found that microbiota compositions were much more similar within strains than between strains (*P* < 0.001 for unweighted and weighted UniFrac) (Fig. 1B; Supplemental Fig. 1). However, because the members of an inbred strain share a recent common ancestor, it is unclear to what extent the shared microbiota result from parent to offspring transfer of microbiota as compared to host genetic factors. The standard ways to estimate heritability, defined in an outbred population as the proportion of the phenotypic variance contributed by the genetic variance (Falconer and Mackay 1996; Lynch and Walsh 1998), are to examine pedigrees or compare monozygotic with di-zygotic twins. In mice, this is traditionally done using inter-crosses between strains differing in traits of interest. Because of the problem of maternal seeding, we estimated heritability by a different method, based on the proportion of phenotype variance accounted for by genetic relationships among the strains.

All of the HMDP strains have been either sequenced or densely genotyped (http://www.jax.org), allowing us to determine their genetic relatedness. Based on this SNP-based approach (rather than a family-based approach), we were able to estimate the heritability of the abundance of the major taxa of gut microbiota (Supplemental Table 3). For the calculation, we utilized a linear mixed model and assumed additive effects (see Methods). The assumption behind the linear mixed model approach is that the covariance of the genetic component of the phenotypic data is proportional to the kinship matrix or genetic similarity matrix between the animals. In this model, each individual mouse microbiome composition (relative abundance of each taxa) is affected by a genetic random effect, which is correlated across mice by virtue of sharing some of the genetic variants affecting microbiome, and an environmental random effect, which is uncorrelated across mice.

When maintained under controlled conditions, host genetic variation appears to explain a substantial amount of the variation in gut microbiota composition in the HMDP, up to 0.5 or more for many common taxa (Table 1; Supplemental Table 3). The range of heritabilities of microbiota was similar for phyla, families, and genera, and for males and females (Supplemental Table 3) and approached the range of heritabilities we observed for measured clinical phenotypes (Supplemental Table 4).

**Table 1.**
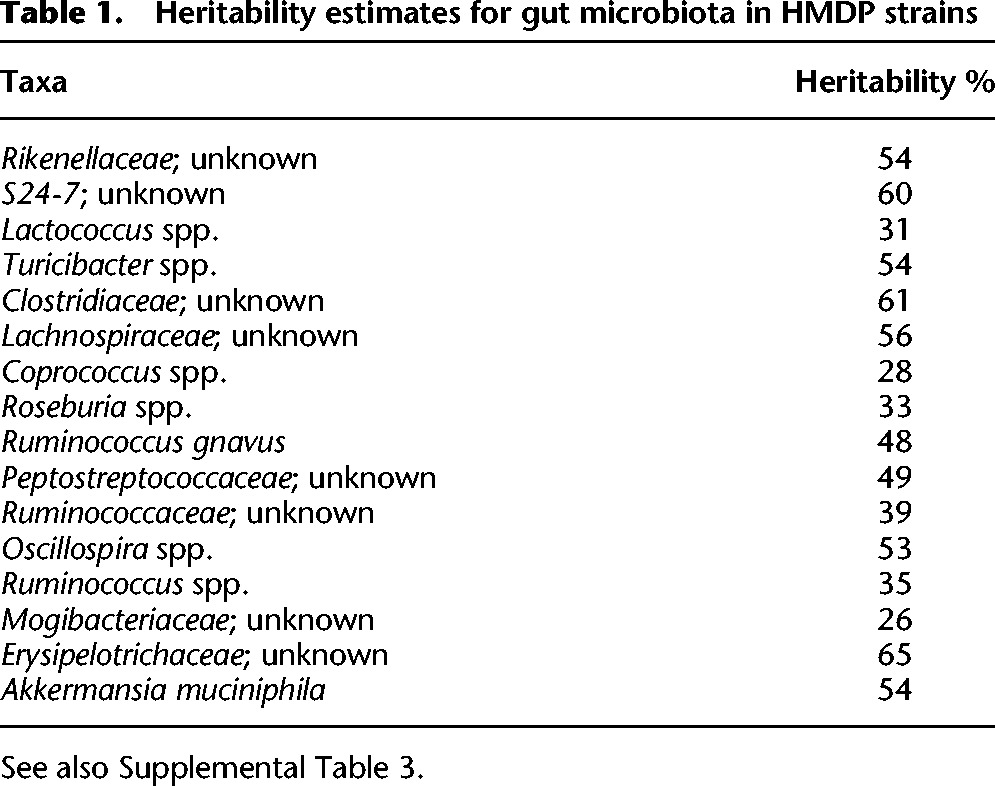
Heritability estimates for gut microbiota in HMDP strains

Our approach for estimating the heritability of gut microbiota composition is potentially confounded by the complication of physical transmission (Ubeda et al. 2012; McCafferty et al. 2013). However, since most of the inbred strains have been separated for many decades, it seems unlikely that a particular composition would be maintained over so many generations if it was due largely to physical “seeding.” A second possible caveat in this analysis is that multiple animals from the same strain were, in some cases, housed in the same cage, and they may share similar microbiota compositions due to physical transfer rather than host genotype. We rule out this confounding factor by performing the same analysis using only one animal per strain (Supplemental Table 3). We expected lower heritability than in the complete cohort because of reduced total genetic relatedness and power. However, even in the reduced sample, while our estimates are lower than the complete data set, host genotype still accounts for a substantial fraction of the variation.

### Gut microbiota contribute to dietary responsiveness

Among the HMDP strains examined were striking differences in response to the HF/HS diet. Some strains showed as much as a sixfold increase in body fat whereas others showed no significant change, and food consumption was only modestly associated with the gain in body weight (*r*^2^ = 0.30) or the gain in fat mass (*r*^2^ = 0.04) (Parks et al. 2013). Likewise, HOMA-IR, a measure of insulin resistance, showed over a 50-fold range among the strains (Parks et al. 2015). We hypothesized that the composition of the gut microbiota might contribute to this variation.

**Figure 2.**
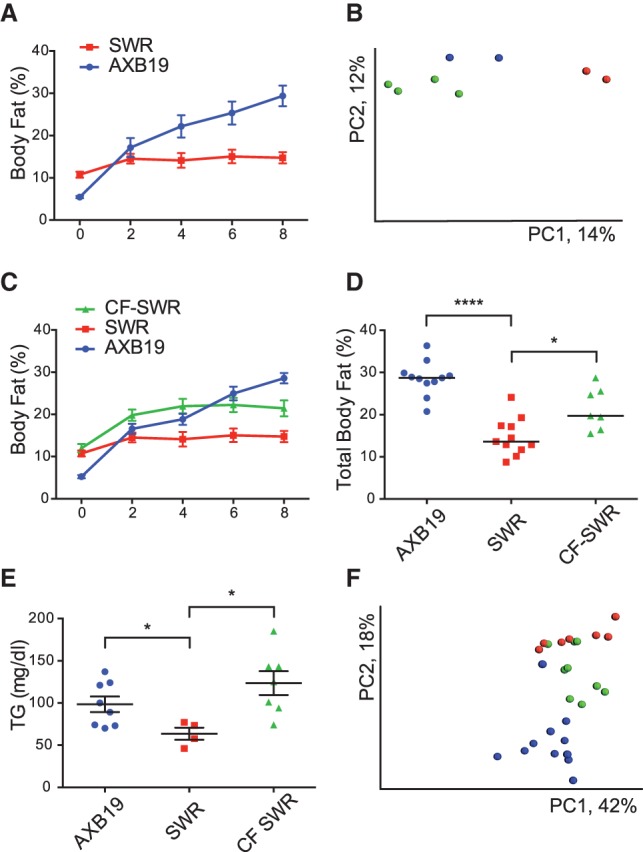
Cross-fostering influences dietary responsiveness. (*A*) Body fat increase in SWR and A×B19 strains during 8 wk of the HF/HS diet. (*B*) Principal Coordinate Analysis (PCoA) of unweighted UniFrac distances for fecal samples after cross-fostering newborn SWR and A×B19 pups between parents (blue = A×B19 mothers, red = SWR mothers, and green = cross-fostered SWR pups), *P* < 0.05 for unweighted UniFrac using Student's *t*-test with 1000 Monte Carlo simulations. (*C*) Body fat changes after cross-fostering SWR pups with A×B19 mother (CF-SWR) compared to SWR and A×B19 controls. (*D*) Total body fat percentage after 8 wk of HF/HS feeding. (*E*) Plasma triglycerides (TG) levels. (*F*) PCoA of unweighted UniFrac distances for cecum samples after 8 wk of the HF/HS diet (blue = A×B19, red = SWR, green = cross-fostered SWR). Significant differences: (*) *P* < 0.05, (****) *P* < 0.0001, according to unpaired two-tailed Student's *t*-test. See also Supplemental Figure 2.

To test the hypothesis, we performed neonatal cross-fostering experiments between two strains, A×B19 and SWR, exhibiting diverse responses after HF/HS feeding. Figure 2A shows the response of the two strains to the diet in terms of body fat increase during 8 wk of HF/HS diet feeding. The A×B19 strain gained ∼24 g fat in response to the diet while strain SWR gained ∼4 g (Fig. 2A; Supplemental Fig. 2A). We cross-fostered newborn SWR mice with A×B19 dams and observed that the gut microbiota composition of the cross-fostered pups resembled A×B19 mice rather than SWR mice, indicating efficient transfer (Fig. 2B). At 4 wk of age, we placed the pups on the HF/HS diet and monitored fat gain. In a pilot study (Supplemental Fig. 2B), both cross-fostered male and female mice initially exhibited increased weight gain as compared to SWR mice. While the male mice continued to show an increased response up to 8 wk on the diet, the female mice became similar to SWR mice after 8 wk. We then repeated the study with a larger group of cross-fostered mice (*n* = 8–11 per group). Again, the male cross-fostered mice showed significantly more weight gain and higher body fat composition (*P* < 0.01) as compared to SWR control mice (Fig. 2C,D). In addition, cross-fostered male SWR mice also showed higher levels of plasma triglyceride compared to SWR mice (Fig. 2E). The female mice, on the other hand, did not exhibit a significant increase in body fat at 8 wk of age (Supplemental Fig. 2C). After 8 wk of the HF/HS diet, the microbiota composition of the cross-fostered SWR pups moved back toward SWR microbiota composition, supporting the role of host genotype in microbiota community structure (Fig. 2F). We conclude that common variations in the composition of the gut microbiome contribute in part to the response to a HF/HS diet.

### Gut microbiota associations with metabolic and cardiovascular traits

To identify which bacteria contribute to obesity and metabolic phenotypes, we sought to identify potential relationships between metabolic traits and the gut microbiota. Altogether, we identified many correlations, including some novel and some known relationships (Supplemental Table 5). Several of these appear to be significant, based on a false discovery rate of <0.01.

Two taxa from the family Lachnospiracea, *Roseburia* spp. and *Ruminococcus gnavus*, were positively associated with obesity and metabolic traits including body fat increase on a HF/HS diet, insulin levels, and HOMA-IR (*P* < 0.001). The same traits were also associated with an unknown species of *Lactobacillus* (Supplemental Table 5). Our data are consistent with a recent study, showing that increased abundance of *Roseburia* spp. in obese subjects is positively correlated with body mass index and inflammation (Tims et al. 2013; Verdam et al. 2013), and *Lactobacillus reuteri* has been previously linked to increased obesity in humans (Million et al. 2012). *A. muciniphila* was inversely correlated with body fat (*r* = −0.15; *P* = 9.02 × 10^−4^) and insulin levels (*r* = −0.20; *P* = 4.57 × 10^−6^) (Supplemental Table 5). *A. muciniphila* is a mucin-degrading, gram-negative anaerobe residing in intestinal mucus layers that has been associated with obesity and insulin resistance in humans and mice (Derrien et al. 2011; Everard et al. 2013).

### *Akkermansia muciniphila* treatment improves obesity and metabolic parameters in mice fed a high-fat/high-sucrose diet

**Figure 3.**
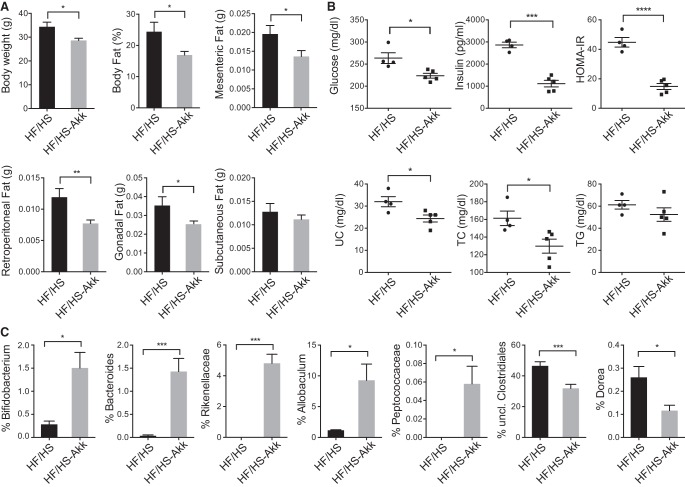
*Akkermansia muciniphila* treatment reduces obesity and metabolic syndrome traits in mice fed a HF/HS diet. (*A*) Total body weight, body fat, mesenteric, retroperitoneal, gonadal, and subcutaneous fat depot weights (g per 100 g body weight) in mice treated by oral gavage with live or heat-inactivated *A. muciniphila* and fed a HF/HS diet (*n* = 5). (*B*) Glucose, insulin, HOMA-IR, unesterified cholesterol (UC), total cholesterol (TC), and triglycerides (TG) levels. (*C*) Relative abundance of bacterial genera between different treatment groups. Data are shown as means ± SD. Significant differences: (*) *P* < 0.05, (**) *P* < 0.01, (***) *P* < 0.001, (****) *P* < 0.0001, with unpaired two-tailed Student's *t*-test.

To test causality of the relationship, we administrated live or heat-killed *A. muciniphila* to obesity-prone A×B19 male mice (Supplemental Fig. 3A). Ten-week-old male A×B19 mice were treated five times per week with *A. muciniphila* by oral gavage at a dose of 1.44 × 10^9^ cfu/0.2 mL (HF/HS-Akk), while control mice were treated with an oral gavage of an equivalent volume of heat-killed *A. muciniphila* (HF/HS). After 1 wk of gavage, all mice were put on HF/HS diet for four additional weeks. After 5 wk of gavage, we observed that mice given *A. muciniphila* showed significantly improved metabolic parameters. Figure 3A shows that body weight and total body fat, including all fat depots examined, were significantly reduced in *A. muciniphila*-treated mice. Plasma lipid levels showed substantial decreases in total cholesterol and triglycerides (Fig. 3B). Most striking were the effects on insulin resistance, with dramatically decreased levels of both glucose and insulin (Fig. 3B). Our data are consistent with the correlations observed among the HMDP as well as recent findings (Everard et al. 2013).

In addition to metabolic changes, the administration of *A. muciniphila* altered the gut microbiota composition (Fig. 3C). Thus, using both chow and HF/HS diets, *A. muciniphila*-treated mice clustered separately from mice that received heat-inactivated bacteria (Supplemental Fig. 3A,B). This difference was evident at the phylum level, showing significant shifts between two dominant phyla, Bacteoridetes and Firmicutes (Supplemental Fig. 3C). Surprisingly, we did not observe significant differences in the total abundance of *A. muciniphila*, perhaps because DNA from heat-killed bacteria was also present.

### Genome-wide association (GWAS) analysis of loci controlling gut microbiota in mice

**Figure 4.**
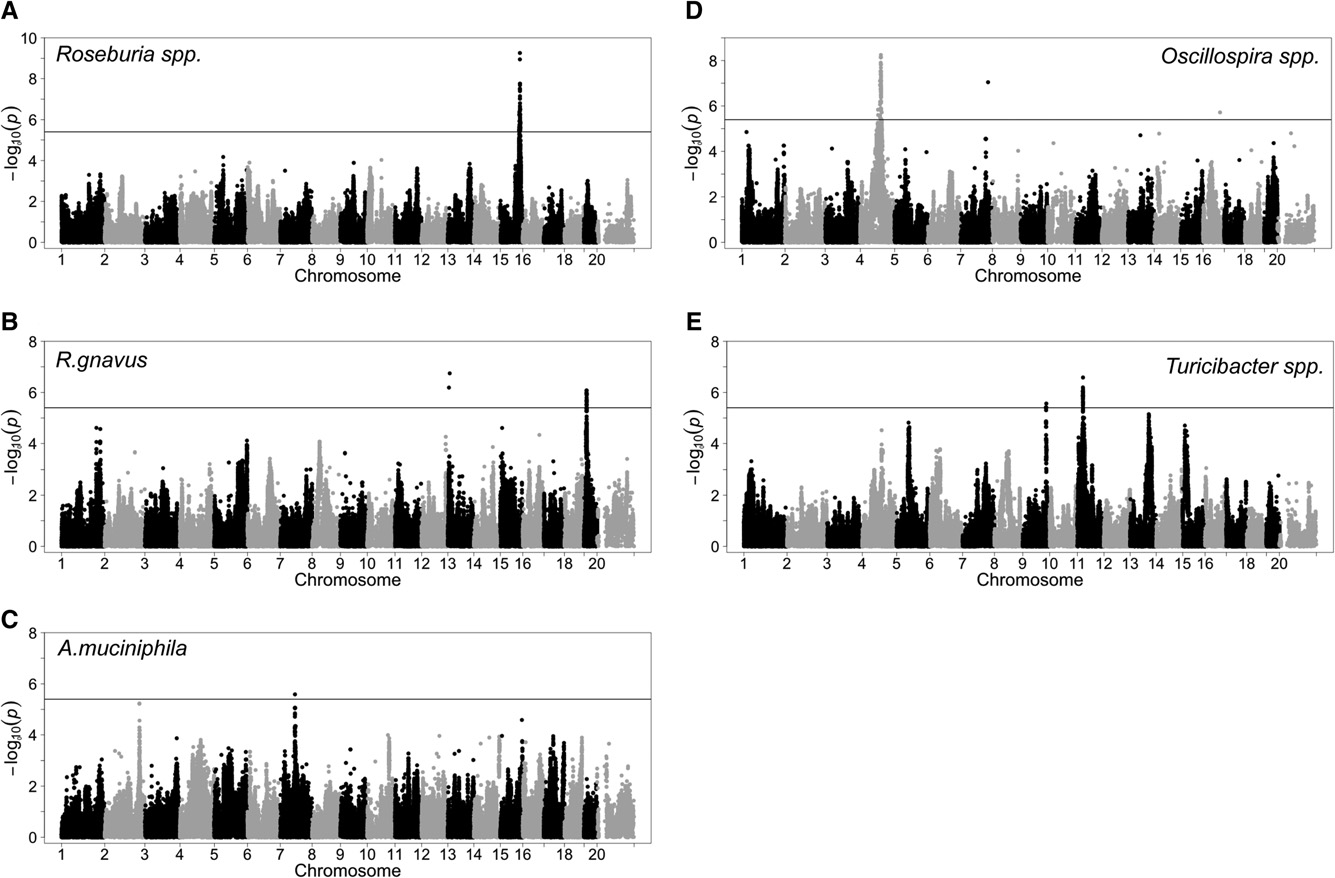
Genome-wide association mapping of gut microbiota genera in the HMDP: (*A*) *Roseburia* spp., (*B*) *R. gnavus*, (*C*) *A. muciniphila*, (*D*) *Oscillospira* spp., (*E*) *Turicibacter* spp. Association was performed using the FaST-LMM algorithm (Lippert et al. 2011) following correction for population structure using ∼200,000 filtered SNPs genotyped in all strains. The horizontal line indicates the threshold for genome-wide significance (*P* < 4 × 10^−6^). See also Supplemental Table 6.

Next, we aimed to obtain evidence for specific interactions between gut microbiota and host genetics. Rather than using linkage analysis, as is traditional in mouse genetics, we employed association across the HMDP strains, since the resolution of mapping is one or two orders improved (Bennett et al. 2010). Such association analysis has now been used to identify novel genes which were subsequently validated in a number of cases (Farber et al. 2011; Lavinsky et al. 2015; Parks et al. 2015; Rau et al. 2015), but given the structure of the inbred mouse population, there is some potential for long-range linkage disequilibrium. The proportion of each common taxon was treated as an individual trait, and association analyses were performed with 198,431 informative SNPs spaced throughout the mouse genome using a mixed-model algorithm that corrects for population structure (Kang et al. 2008). The threshold for genome-wide significance was based on simulation and permutations as previously described (Farber et al. 2011). Altogether, seven genome-wide significant loci (*P* < 4 × 10^−6^) were found to be associated with common genera (Fig. 4; Supplemental Table 6). Loci ranged from 800 kb to 3 Mb in size and in most cases contained several genes within a linkage disequilibrium block. The majority of these significant associations were detected with members of the classes Clostridia (Lachospiraceae, Ruminococcacea, and Bacilli), and most exhibited similar associations in both sexes (Supplemental Table 6). In order to test whether the GWAS results were inflated by the effect that multiple animals from the same strain were housed in the same cage, we performed GWAS using only one animal per strain. Even with the reduced sample size, we were able to detect GWAS associations in the same regions (Supplemental Table 6), albeit with reduced significance. We have carried out expression profiling of adipose and liver of the HMDP strains when maintained on HF/HS diets and used the data to identify *cis* expression quantitative trait loci (eQTL). These provide a useful means of prioritizing candidate genes at the relevant loci since they provide evidence of functional variation (Civelek and Lusis 2014). The significant *cis*-eQTLs at each of the GWAS loci are shown in Supplemental Figure 5 and Supplemental Table 7. Here, we focus on those genera that show strong correlation with clinical traits, as discussed above; additional loci are described in detail in the Supplemental Material.

**Figure 5.**
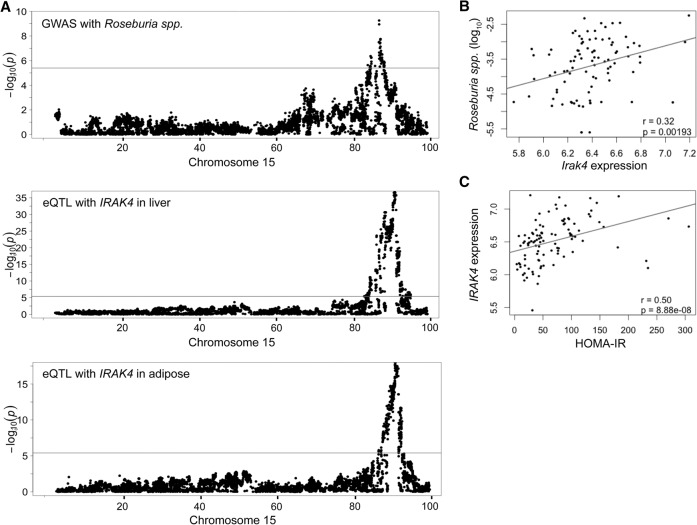
Chromosome 15 locus for abundance of genus *Roseburia* spp. (*A*) Overlapping genome-wide significant associations with the abundance of *Roseburia* spp. and liver and adipose eQTLs of the *Irak4* gene in HMDP mice fed a HF/HS diet. The horizontal line indicates the threshold for genome-wide significance (*P* < 4 × 10^−6^). (*B*,*C*) Correlations of *Irak4* adipose gene expression with the relative abundance of *Roseburia* spp. and HOMA-IR in the HMDP mice. (*r*) Biweight midcorrelation, (*p*) *P* value. See also Supplemental Tables 6 and 7.

For *Roseburia* spp., we identified significant associations spanning 2.6 Mb on Chromosome 15 (Fig. 4A; Supplemental Table 6). The same region showed a significant association with subcutaneous fat mass on a HF/HS diet (*P* < 10^−7^) (Supplemental Fig. 4A), a clinical trait that is also positively correlated with the abundance of *Roseburia* spp. (*r* = 0.25, *P* = 3.9 × 10^−10^) (Table 2). Global gene expression in epididymal adipose tissue and liver showed a significant *cis*-eQTL between the peak SNP (rs31843241) and transcript levels of the *Kif21a*, *Lrrk2*, and *Irak4* genes (Table 2; Fig. 5A; Supplemental Fig. 5; Supplemental Table 7). The expression of *Irak4*, a gene involved in the initiation of the innate immune response, was correlated with the abundance of *Roseburia* spp. and HOMA-IR, suggesting a causal relationship (Fig. 5B,C).

**Table 2.**
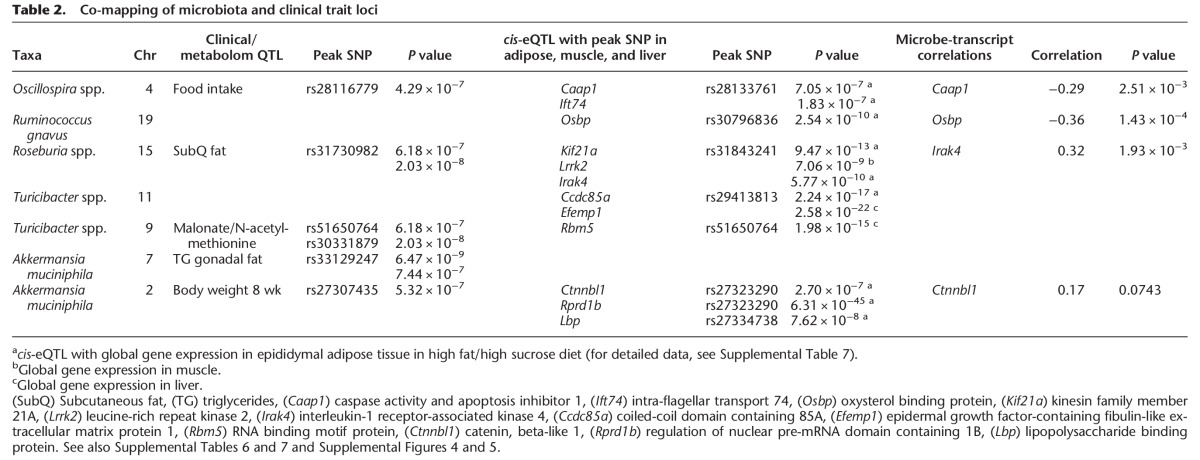
Co-mapping of microbiota and clinical trait loci

*Ruminococcus gnavus* exhibited genome-wide significant association to a locus on Chromosome 19 (Fig. 4B; Supplemental Table 6). The peak SNP (rs30796836, *P* = 8.37 × 10^−7^) has a significant *cis*-eQTL with the transcript levels of the *Osbp* (oxysterol binding protein) gene in adipose tissue of HF/HS-fed mice (females: *P* = 1.51 × 10^−12^; males: *P* = 2.54 × 10^−10^), and the abundance of *Ruminococcus gnavus* negatively correlated with expression of *Osbp* (*r* = −0.36; *P* = 0.00014) (Table 2; Supplemental Fig. 5; Supplemental Table 7).

**Figure 6.**
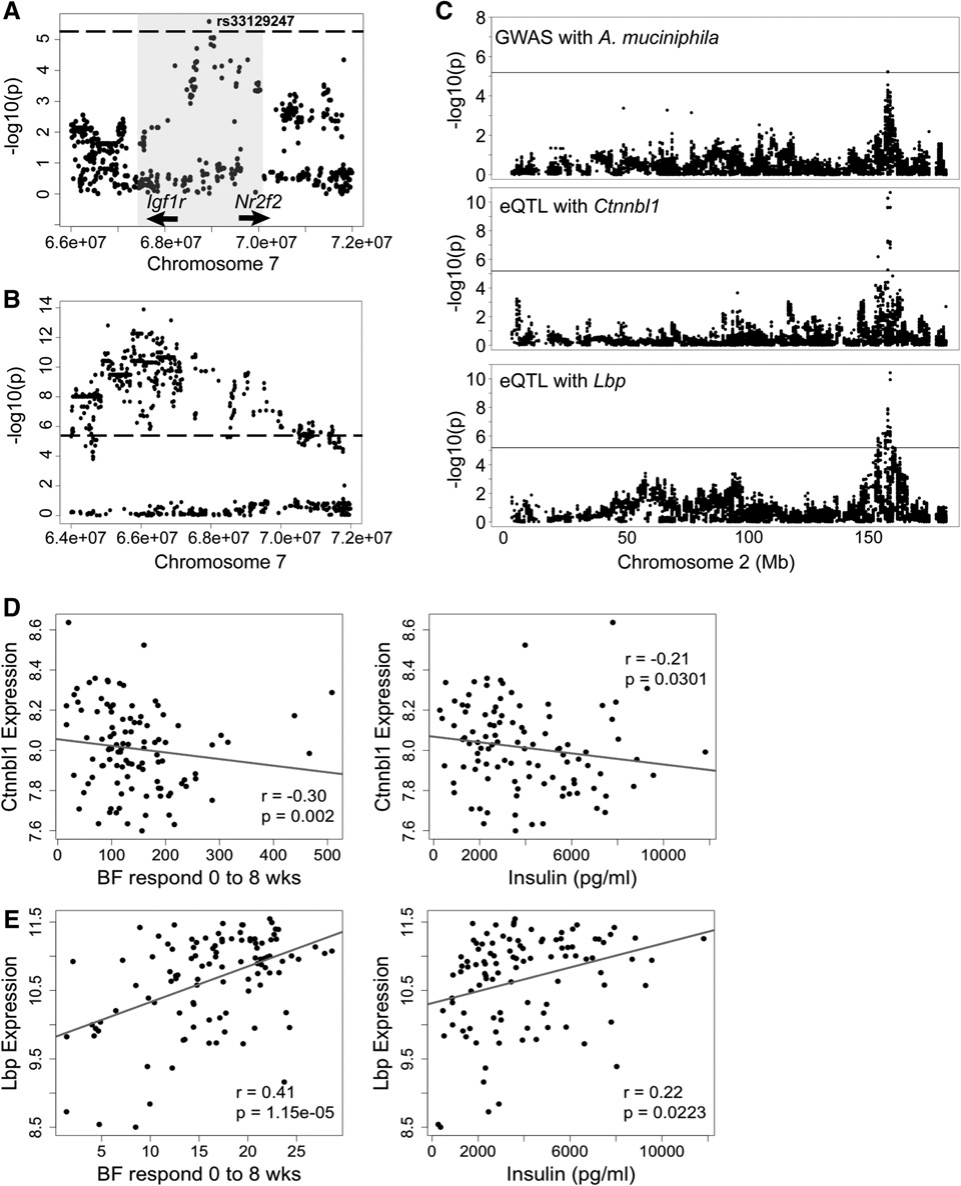
Chromosome 2 and 7 loci for abundance of *A. muciniphila*. (*A*) Locus plot for genome-wide significant association of *A. muciniphila* abundance to a Chromosome 7 locus, indicating the LD block (shaded in gray) and peak SNP rs33129247. Locations of candidate genes are indicated. (*B*) Locus plot for association with TG (triglyceride) at the Chromosome 7 locus. See also Supplemental Tables 6 and 7. (*C*) Chromosome 2 locus showing overlapping associations with the abundance of *A. muciniphila* and *cis*-eQTLs of the *Ctnnbl1* and *Lbp* genes in adipose. (*D*) Correlation of epididymal adipose gene expression of *Ctnnbl1* with body fat and insulin levels after 8 wk of the HF/HS diet. (*E*) Correlation of epididymal adipose gene expression of *Lbp* with body fat response and insulin levels after 8 wk of the HF/HS diet. (BF) Body fat, (*r*) biweight midcorrelation, (*p*) *P* value.

Finally, we detected significant associations for the abundance of *A. muciniphila* on Chromosomes 7 (rs33129247; *P* = 2.59 × 10^−6^) and 2 (rs27323290, *P* = 6.67 × 10^−6^) (Figs. 4C, 6A; Supplemental Table 6). The peak SNP on Chromosome 7 (rs33129247) was also associated with triglyceride levels (*P* = 6.47 × 10^−9^) and gonadal fat (*P* = 7.44 × 10^−7^) (Table 2; Fig. 6B; Supplemental Fig. 4B). Strong candidates for this locus are *Igf1r* and *Nr2f2* genes, since both have been shown to play a role in glucose and insulin regulation (Ueki et al. 2006; Garg et al. 2011). The Chromosome 2 locus contains *Ctnnbl1*, a gene implicated in obesity (Liu et al. 2008; Tan et al. 2014) and two interesting candidates, bactericidal/permeability-increasing protein (*Bpi*) and lipopolysaccharide binding protein (*Lpb*). *Ctnnbl1* also showed a significant association with food intake (*P* = 1.17 × 10^−9^) and total weight after 8 wk on the HF/HS diet (*P* = 5.8 × 10^−8^) (Table 2). The *Lbp* and *Ctnnbl1* genes both have significant *cis*-eQTLs in adipose and liver (Table 2; Fig. 6C; Supplemental Fig. 5; Supplemental Table 7) and are associated with body fat percentage increase and insulin levels (Fig. 6D,E).

## Discussion

We previously showed that inbred strains of mice differ dramatically in their response to a high-fat, high-sucrose diet (Parks et al. 2013). Based on the large body of evidence indicating that gut microbiota can influence metabolic traits (Backhed et al. 2004, 2007; Turnbaugh et al. 2006, 2009; Ridaura et al. 2013), we hypothesized that the dietary response was dictated in part by differences in gut microbiota. We showed that different inbred strains differ strikingly in the composition of gut microbiota and provided evidence that the variation is determined in part by the host genetic background. Consistent with our hypothesis, we showed that cross-fostering between two strains of mice affected dietary response to the high-fat, high-sucrose diet. By correlating microbiota composition with dietary response among the HMDP inbred strains, we were able to identify several candidate microbiota influencing dietary response. We chose one of these, *A. muciniphila*, to examine using gavage with the cultured microbe and observed striking effects on weight gain, adiposity, plasma lipids, and insulin resistance. Finally, to help identify novel host-microbiota interactions, we have mapped loci controlling certain microbiota taxa. We discuss each of these findings in turn below.

Experimental studies have shown that the host genetic background can influence gut microbiota composition. For example, mice with mutations affecting inflammatory signaling or diabetes differ in microbiota composition from their wild-type littermates (Henao-Mejia et al. 2012; Peng et al. 2014). However, the importance of common genetic variants in contributing to the composition of the gut microbiota is unclear. While twin pairs and related individuals share gut microbiota to a greater extent than unrelated individuals, early studies did not find a statistically significant difference in gut microbiota sharing in monozygotic (MZ) and dizygotic (DZ) twins. However a recent study with 416 twin pairs demonstrated that MZ twins have greater overall microbial community similarities than DZ twin pairs and identified several microbial taxa with relative abundances that differ depending on host genetics (Goodrich et al. 2014). Studies using genetic crosses of mice, where the environment can be controlled, also suggest that host genetics can significantly alter gut microbiota composition (Benson et al. 2010; McKnite et al. 2012; Srinivas et al. 2013).

Heritability represents the fraction of variation that is attributable to genetic variation and is a relative value that depends on the environment and the degree to which the population varies. Traditionally, heritability has been estimated using pedigrees in outbred populations or by comparing monozygotic versus di-zygotic twins. In mice, heritability is generally estimated by analyzing genetic crosses, but for studies of gut microbiota, this is confounded by the fact that there is physical transmission of “seed” microbiota from generation to generation (Ubeda et al. 2012). To circumvent this problem, we used a SNP-based approach to determine relatedness (Yang et al. 2010) rather than a family-based approach. All of the inbred strains constituting the HMDP are separated from one another by many generations (Silver 1995) and thus are unlikely to share microbiota as a result of physical transmission. Our results indicate a high degree of heritability of the major groups of microbiota in mice, ranging from ∼0.3 to >0.5, although we note certain caveats in our approach (see Results). This high heritability presumably results from the fine tuning of a symbiotic relationship that has co-evolved for millions of years. Among the likely contributing factors are differences in immunoglobulin and antibacterial molecules secreted into the gut lumen (Wen et al. 2008; Vijay-Kumar et al. 2010; Shulzhenko et al. 2011), differences in the mucosal gut structure (Sommer et al. 2014; Wlodarska et al. 2014), and differences in bile acid metabolism (Ryan et al. 2014).

Previous studies have shown that certain large effect mutations affecting inflammatory signaling or metabolic traits can significantly affect microbiota composition and that, in some cases, these can be transmitted by transplantation of gut microbiota from such mice (Henao-Mejia et al. 2012; Peng et al. 2014). Here, we have examined whether common variations in gut microbiota are also causally involved in metabolic traits. To test this possibility, we chose two inbred strains, A×B19 and SWR, that differ strikingly in the response to a HF/HS diet for cross-fostering studies. When male SWR mice cross-fostered by A×B19 dams were subjected to the HF/HS diet, they gained ∼8% in total body fat as compared to ∼2% for SWR, while A×B19 gained ∼25% (Fig. 2C). Thus, while the majority of the response was dictated by the host genetic background, the gut microbiota did contribute significantly. Human gut microbiota exhibit more diversity and quantitative variation than what we have observed among common mouse strains, suggesting that a significant fraction of variance in obesity and insulin resistance in human populations is due to microbiota composition. Conceivably, this could explain some of the “missing heritability” observed in GWAS. We used correlation analysis to identify candidate microbiota contributing to the response to the HF/HS diet. Several genera were found to be strongly associated with traits such as body fat, plasma lipids, and insulin resistance, including some, such as *Roseburia* spp. and *A. muciniphila*, that have previously been implicated in metabolic traits. We chose *A. muciniphila*, a mucin-degrading, gram-negative anaerobe, to examine its potential response to our dietary challenge. We administered by oral gavage either live or heat-killed bacteria to strain A×B19 male mice for 1 wk and then began the HF/HS challenge, continuing to administer the live or heat-killed bacteria for a total of 5 wk. Significant differences were observed between the groups in body fat gain, plasma lipid levels, and insulin resistance. We noted significant correlations between the abundances of certain taxa across the panel of strains (data not shown), providing information about microbiota community interactions.

Finally, we used the HMDP to perform high-resolution mapping of loci contributing to microbiota abundance. Using association analysis, we identified seven significant loci for five out of 17 common genus level taxa. Most of the loci were observed in both males and females, supporting the conclusion that they are true positives. The loci contain a number of strong candidate genes based on the literature, functional variants, and correlations with clinical and molecular traits (see Supplemental Material). The Chromosome 15 locus for *Roseburia* spp. contains the *Irak4* gene, which is involved in signaling innate immune responses from Toll-like receptors (Flannery and Bowie 2010; Liu et al. 2011). Mice deficient in *Irak4* expression are more susceptible to viral and bacterial infections (Suzuki et al. 2002), and *Irak4* has previously been associated with gut microbiota composition in a subset of BxD RI strains (McKnite et al. 2012). The *A. muciniphila* locus in Chromosome 2 contains two closely linked genes, *Bpi* and *Lbp*. Both bind to bacterial lipopolysaccharide (LPS) and elicit immune responses by presenting LPS to CD14 and TLR4 and signaling the acute-phase immunological response (Muta and Takeshige 2001). *Bpi* acts as an endogenous antibiotic protein with potent killing activity against gram-negative bacteria (Wittmann et al. 2008).

Our data constitute a resource for the further dissection of mechanistic host-gut microbiota interactions. We have identified a number of highly significant associations between gut microbiota and clinical traits, and the loci reported here provide a means of identifying novel host factors controlling gut microbiota abundances.

## Methods

### Sample collection and study design

All mice were obtained from The Jackson Laboratory and were bred at UCLA for two or more generations for use in this study. Briefly, until 8 wk of age mice were maintained on a chow diet (Ralson Purina Company) and then placed on a high-fat, high-sucrose diet (Research Diets D12266B) for an additional 8 wk (Parks et al. 2013). Samples were obtained from the cecum of 599 mice from 113 strains, with an average of six mice per strain (327 males and 297 females) (Supplemental Table 1). Mice from different strains and genders were housed in separate cages but in the same room throughout the study. Cecum and fecal samples were snap-frozen with liquid nitrogen and stored at −80°C. The animal protocol for the study was approved by the Institutional Care and Use Committee (IACUC) at the University of California, Los Angeles.

### Sample preparation and sequencing of 16S rRNA genes

Microbial DNA was extracted using the PowerSoil DNA Isolation Kit (MO BIO Laboratories). Amplification and sequencing of the V4 hypervariable region of the 16S rRNA gene was performed using the validated, region-specific bacterial primers 515F and 806R according to previously described methods (Caporaso et al. 2012) optimized for the Illumina MiSeq platform. The reverse amplification primer contained a 12-bp Golay error-correcting barcode sequence, and amplicons were generated in triplicate using 5 Prime Hot MasterMix (Fischer Scientific). The PCR conditions consisted of an initial denaturation step of 94°C for 3 min; 35 cycles of 94°C for 45 sec, 50°C for 30 sec, and 72°C for 90 sec, followed by 72°C for 5 min. Replicate amplicons were quantified with a Quant-iT PicoGreen dsDNA Assay kit (Life Technologies), pooled (200 ng from 96 samples), and purified using with the UltraClean PCR Clean-up kit (MO BIO Laboratories). High-throughput sequencing analysis of bacterial rRNA genes was performed on the purified, pooled sample using the Illumina MiSeq platform (Illumina).

De-multiplexing 16S rRNA gene sequences, quality control, and operational taxonomic unit binning were performed using the open source pipeline Quantitative Insights Into Microbial Ecology (QIIME) version 1.7.0 (Caporaso et al. 2010; Bokulich et al. 2013). The total number of sequencing reads was 13,805,813 (an average of 23,048 reads per sample) with an average length of 153-bp reads. Sequences were binned into OTUs based on 97% identity using UCLUST (Edgar 2010) against the Greengenes reference database (McDonald et al. 2012). Each sample's sequences were rarefied to 7000 reads to reduce the effect of sequencing depth. Seven samples were omitted from further analysis due to insufficient sequence coverage, yielding 592 samples.

Microbial composition at each taxonomic level was defined using the summarize_taxa function in QIIME. Prior to genome-wide association analysis, taxa at any taxon present in fewer than 75% of samples was discarded, yielding a total of 43 common taxa from different taxonomic levels (three phyla, five classes, six orders, 12 families, and 17 genus level taxa). The relative abundance of each taxon was calculated by dividing the sequences pertaining to a specific taxon by the total number of bacterial sequences for that sample.

### Heritability calculations

Heritability was estimated using a linear mixed with the EMMAX software model (Kang et al. 2010). In this approach, the phenotypes (in this case, the relative abundances) are assumed to be generated by genetic and environmental components. The assumption behind the linear mixed model approach is that the covariance of the genetic component of the phenotypic data is proportional to the kinship or genetic similarity matrix between the animals. The analysis provides estimates of σ_*g*_^2^ and σ_*e*_^2^, the variances corresponding to the genetic and environmental component, respectively. The heritability is then the fraction of the variance accounted for by the genetics or

$$\frac{\sigma_{g}^{2}}{\sigma_{g}^{2}+\sigma_{e}^{2}}$$

and is computed for each relative abundance. We note that the kinship matrix must be standardized for these estimates to be consistent with the classical definition of heritability (Kostem and Eskin 2013; Speed and Balding 2015). A standardized kinship matrix has a mean along the diagonal of 1 and a sum of 0.

### Clinical traits

Body composition, food intake, and blood and plasma assays were as previously described (Parks et al. 2013, 2015). Briefly, mice were measured for total body fat mass and lean mass using nuclear magnetic resonance (NMR) using the Bruker minispec with software from Eco Medical Systems. Blood was collected from mice following fasting for 4–5 h, and plasma was isolated by centrifugation in Microtainer tubes with EDTA (Becton Dickinson). Plasma glucose levels were measured using a Beckman Glucose Analyzer 2 (Beckman Instruments). Plasma total cholesterol, HDL cholesterol, free cholesterol, triglycerides, and free fatty acid concentrations were determined by enzymatic assays employing colorimetric endpoints as described previously (Hedrick et al. 1993). Insulin levels and HOMA-IR were determined as previously described (Castellani et al. 2008).

### Cross-fostering study

Within 24 h of birth, the pups from SWR females were removed from birthing cages and placed with A×B19/PgnJ mothers. Pups were weaned on postnatal day 21, and at 8 wk of age mice were placed on a high-fat/high-sucrose diet (Research Diets D12266B) for an additional 8 wk. Controls from both strains were fostered with different mothers from the same strain.

### *Akkermansia muciniphila* gavage

*A. muciniphila* (ATCC BAA-835) was grown in a Columbia broth medium supplemented with 0.05 % hog gastric Mucin type III (Sigma) under anaerobic conditions (Ganesh et al. 2013). Cells were harvested in late logarithmic phase by centrifugation at 6000 rpm in a tabletop Fisher centrifuge at room temperature and resuspended in 0.05 volume sterile anaerobic PBS containing 25% glycerol to a concentration of 7.2 × 10^10^ per mL prior to storage at −80°C. For gavage, anaerobic cell suspensions were diluted 10-fold in PBS. The sterile anaerobic PBS (pH 7) was supplemented with 0.05% cysteine HCl, degassed with N_2_, sealed in serum bottles with butyl rubber stoppers under anaerobic conditions provided by a gas phase of 1.8 atm N_2_/CO_2_ (80:20, vol/vol).

*A. muciniphila* was administrated to 10-wk-old A×B19 male mice (*n* = 5 per group), housed in groups of 2–3 mice per cage, by oral gavage at a dose 1.44 × 10^9^ cfu/0.2 mL suspended in sterile anaerobic PBS (HF-Akk). Treatment was 5 d per week for 5 wk. Control groups were treated with an equivalent volume of heat-inactivated *A. muciniphila*. After the first week of *A. muciniphila* treatment, all mice were placed on the HF/HS diet for an additional 4 wk.

### Association analyses

Association analyses of taxa were performed using the Factored Spectrally Transformed Linear Mixed Models (FaST-LMM) algorithm adjusting for population structure and using gender as a covariate (Kang et al. 2008; Lippert et al. 2011). To achieve a normal distribution, the sequence counts for each taxonomic bin were Arcsine-transformed. A total of 198,431 informative SNPs (minor allele frequency > 5%; missing genotype rate < 10%) spaced throughout the genome were used. For genome-wide significance, we used a *P* value threshold < 4 × 10^−6^, based on permutation and simulation and which roughly corresponds to a Bonferroni correction (Bennett et al. 2010; Farber et al. 2011). In some cases, multiple animals from the same strain were housed together, and in order to rule out the possibility that GWAS results were inflated because strains shared similar microbiota compositions due to physical transfer, we also performed GWAS choosing a single sample from each strain at random (*n* = 113). Linkage disequilibrium (http://pngu.mgh.harvard.edu/~purcell/plink/) boundaries were determined by calculating SNP correlations and visualizing *r*^2^ > 0.8 in Haploview.

### Expression QTL analysis

To help identify candidate genes at loci associated with taxa abundances, we carried out global expression analysis of epididymal adipose and liver tissue in male and female mice (16 wk old) fed a HF/HS diet as described (Parks et al. 2015). Isolated RNA (two mice per strain) was analyzed for global gene expression using Affymetrix HT_MG-430A arrays and filtered as described (Bennett et al. 2010). Microarray data are available in the Genome Expression Omnibus (GEO; http://www.ncbi.nlm.nih.gov/geo/) under accession number GSE64770. The loci controlling transcript levels were mapped with FaST-LMM and are referred to as expression quantitative trait loci. Loci are defined as *cis* if the peak SNP mapped within 1 Mb of gene position (*P* value threshold < 1.4 × 10^−3^).

### Statistics

All correlations involving bacterial relative abundance were performed using biweight midcorrelation, which is robust to outliers (Wilcox 2005). The statistical cutoff of *P* = 0.1 after false discovery rate (FDR) correction for multiple comparisons was used to define statistical significance for correlations. Statistical analyses were performed using GraphPad Prism. Data are expressed as mean ± SEM, and significance was set at a two-tailed *P* value < 0.05.

## Data access

16S rRNA sequencing data generated for this study have been submitted to the NCBI Sequence Read Archive (SRA; http://www.ncbi.nlm.nih.gov/sra/) under accession number SRP059760. The summary tables for both genders are posted on our website (systems.genetics.ucla.edu/data/hmdp2) with the link called “Download high-fat microbiota vs. clinical trait correlation table” and can also be found in Supplemental Material (Supplemental Table 8).
